# Genetic correlations and little genetic variance for reaction norms may limit potential for adaptation to pollution by ionic and nanoparticulate silver in a whitefish (Salmonidae)

**DOI:** 10.1002/ece3.2088

**Published:** 2016-03-17

**Authors:** Emily S. Clark, Manuel Pompini, Anshu Uppal, Claus Wedekind

**Affiliations:** ^1^ Department of Ecology and EvolutionBiophoreUniversity of Lausanne1015LausanneSwitzerland

**Keywords:** Additive genetic variance, Coregonid, inducible defense, micropollutant, plasticity, rapid evolution

## Abstract

For natural populations to adapt to anthropogenic threats, heritable variation must persist in tolerance traits. Silver nanoparticles, the most widely used engineered nanoparticles, are expected to increase in concentrations in freshwaters. Little is known about how these particles affect wild populations, and whether genetic variation persists in tolerance to permit rapid evolutionary responses. We sampled wild adult whitefish and crossed them in vitro full factorially. In total, 2896 singly raised embryos of 48 families were exposed to two concentrations (0.5 *μ*g/L; 100 *μ*g/L) of differently sized silver nanoparticles or ions (silver nitrate). These doses were not lethal; yet higher concentrations prompted embryos to hatch earlier and at a smaller size. The induced hatching did not vary with nanoparticle size and was stronger in the silver nitrate group. Additive genetic variation for hatching time was significant across all treatments, with no apparent environmental dependencies. No genetic variation was found for hatching plasticity. We found some treatment‐dependent heritable variation for larval length and yolk volume, and one instance of additive genetic variation for the reaction norm on length at hatching. Our assessment suggests that the effects of silver exposure on additive genetic variation vary according to trait and silver source. While the long‐term fitness consequences of low‐level silver exposure on whitefish embryos must be further investigated to determine whether it is, in fact, detrimental, our results suggest that the evolutionary potential for adaptation to these types of pollutants may be low.

## Introduction

Technological innovations can pose significant threats to natural populations if their products or by‐products are released into the environment. A primary concern for conservation biologists is whether or not natural populations will be able to rapidly adapt to these novel anthropogenic stressors. In recent decades, one of the foremost challenges that aquatic organisms have faced is exposure to various toxicants through the effluents of sewage treatment plants (Fabrega et al. 2011; Brazzola et al. 2014). Engineered nanoparticles, that is, materials that have at least one dimension on the nanoscale (≤ 100 nm), are some of the newest and fastest‐rising agents predicted to enter the waste stream and subsequently the aquatic environment (Asghari et al. 2012). Silver nanoparticles (AgNP) are the largest class of nanoparticles used in industrial applications, ranging from textiles to pharmaceutics to electronics (Navarro et al. 2008; Li and Lenhart 2012). Accordingly, AgNP in particular are anticipated to increase in concentrations in natural waters in the coming decade (Handy et al. 2008; Scown et al. 2010b). Current concentrations of AgNP in surface waters are generally estimated to be on the order of 3 × 10^−5^ *μ*g/L to 0.5 *μ*g/L (Scown et al. 2010a,b) or higher. It is notoriously challenging to quantify concentrations in the wild (Mueller and Nowack 2008; Gottschalk et al. 2009), as nanoparticles are prone to undergoing dynamic transformations once in the environment (Scown et al. 2010b; Schultz et al. 2015). Moreover, monitoring of silver residues in sewage influents and effluents was largely suspended in the 1990s (Muth‐Köhne et al. 2013), making it difficult to quantify potential discharge.

The physiochemical properties that render AgNP so versatile and suitable for numerous commercial products also potentially make them hazardous to aquatic organisms. Specifically, the similarity in scale between engineered nanoparticles and natural biomolecules, in addition to their large surface area, increased reactivity, and high mobility, makes them more likely to interfere in biological processes, for example, dynamics of cell membranes or cell‐signaling pathways (Klaine et al. 2012) as well as generate reactive oxygen species and induce oxidative stress (Massarsky et al. 2014). Indeed, a number of investigations into the potential toxicity of AgNP have demonstrated that they can provoke a number of negative effects in aquatic organisms, ranging from sublethal to lethal (Griffitt et al. 2008; Navarro et al. 2008; Scown et al. 2010b; Fabrega et al. 2011; Garcia‐Alonso et al. 2014).

Despite the apparent toxicity of AgNP to aquatic organisms, the impact that these particles may have on members of wild populations under ecologically relevant scenarios remains largely unknown. Most studies to date have utilized model organisms, at least for early developmental stages, and have focused on the effects of acute exposure to AgNP (but see: Mackevica et al. 2015; Sakamoto et al. 2015). Notably, for low‐mobility species or life‐history stages, this type of risk assessment may not be the most appropriate, necessitating longer‐term exposure studies. Moreover, the concentrations used in the majority of studies have been considerably higher than those predicted in the aquatic environment, which are currently on the low microgram to nanogram per liter scale (Mueller and Nowack 2008; Gottschalk et al. 2009). Consequently, there is a need to examine the effects of long exposures to lower concentrations of AgNP on organisms originating from wild populations.

While it is important to assess the effect of AgNP on phenotype, from a conservation perspective, it is also essential to examine whether members of a population vary in their expression of a phenotype and determine whether the variation has a heritable genetic basis. Indeed, if members of a population are unable to avoid stressful ecological conditions or are insufficiently plastic in their responses, then fast adaptation to the novel environment may be the only means of coping and avoiding extinction (Carlson et al. 2014). However, a population's ability to rapidly evolve hinges upon the existence of standing genetic variation for tolerance traits, as it provides the raw material on which selection can immediately act. Whether or not such variation exists in silver tolerance traits has yet to be established in wild populations.

Importantly, as additive genetic variation is not static, but rather highly dependent on environmental conditions (Hoffmann and Merilä 1999), it is equally vital to determine how a particular stressor affects this variation in fitness‐relevant traits. Stress can (1) increase this variation, for example, by lowering the threshold for trait expression and releasing cryptic genetic variation (Gibson and Dworkin 2004; McGuigan and Sgro 2009), (2) decrease it by increasing the environmental component of variation (Charmantier and Garant 2005) or inhibiting an organism from reaching its genetic potential (Merilä and Sheldon 1999), or (3) have neutral effects (Pakkasmaa et al. 2003; Merilä et al. 2004; Clark et al. 2013a). To deepen our understanding of how silver nanoparticle pollution affects the evolutionary potential of wild populations, controlled laboratory experiments comparing additive genetic variation in fitness traits between benign and silver‐treated environments are required.

Here, we have examined how prolonged exposure to low concentrations of AgNP affected development in embryos stemming from a wild population of lake‐dwelling whitefish (*Coregonus palaea*). Coregonids, such as most other salmonids, are keystone species in their respective habitats. They lend themselves to such studies as phenotypic variation is typically considerable for early life‐history traits, such as developmental rate (Thomassen et al. 2011; Skoglund et al. 2012; Vehvilainen et al. 2012) or tolerance to abiotic and biotic stressors (Jensen et al. 2008; Wedekind et al. 2008b), and some of this variation has been found to have a significant additive genetic component (Clark et al. 2013b; Pompini et al. 2013). Moreover, as nanoparticles have the tendency to aggregate and collect at the surface of sediments and substrata (Bradford et al. 2009) where whitefish embryos settle after fertilization, these fish are potentially more exposed than organisms which remain in the water column (Handy et al. 2011, 2012).

Here, we concentrate on a specific whitefish population that spawns near the Bay of Vidy in Lake Geneva. This particular location is considered the most contaminated area of the lake (Loizeau et al. 2004), in terms of micropollutants, as it is the release site of treated and untreated wastewater from Lausanne's municipal treatment plant. Lausanne (population of over 300,000 inhabitants) is the fourth largest in Switzerland, so this whitefish population's exposure to municipal effluents can be assumed to be on the upper range in the country (Brazzola et al. 2014). Sediments in this area have been shown to have markedly higher concentrations of heavy metals in comparison with other regions of the lake (Loizeau et al. 2004). Although concentrations of silver nanoparticles have not specifically been measured in this area, it is assumed that they will accumulate at this location as nanoparticle‐use grows, particularly as the majority of these compounds are released from consumer products into the sewer system and end up in water treatment plants (Li et al. 2013). This population of whitefish may therefore be particularly at risk when it comes to silver nanoparticle exposure.

Adult whitefish were sampled from their spawning site, and their gametes were crossed full factorially to produce all possible sibling combinations. Embryos were then exposed singly to AgNP. As the toxicity of AgNP can vary depending on diameter (Fabrega et al. 2011), we used two sizes of commercially available particles. To determine whether the nanoparticles were more or less harmful than equivalent amounts of ionic silver, a known toxin of aquatic organisms (Erickson et al. 1998), subsets of embryos were also treated with silver nitrate. By virtue of our breeding design, we were then able to separate additive genetic from maternal environmental effects on the expression of early life‐history traits.

## Methods

### Whitefish sampling, embryo rearing, and larval measurements

Large‐type adult whitefish were caught from their spawning grounds in Lake Geneva (Bay of Vidy) with gill nets. Four females and 12 males were haphazardly selected and stripped of their gametes. These gametes were subsequently used for full‐factorial in vitro fertilizations, as described in von Siebenthal et al. (2009), yielding 48 full‐sib families. Embryos were distributed singly to 24‐well plates (Falcon, Becton Dickinson) in a block design, such that two plates contained one replicate set. Each well had previously been filled with 2 mL of aerated and tempered standardized water (OECD 1992). Plates were stored in a 6.5°C temperature‐controlled chamber. Due to variable fertilization success among females, the design was condensed at 136.5 degree days (DD) postfertilization, and embryos were redistributed to 24‐well plates, again such that a set of two plates contained one replicate set. Embryos were thereafter monitored weekly with a light table (LP 555; Hama professional, Monheim, Germany) and a stereo zoom microscope (Olympus SZX9; Olympus Schweiz AG, Volketswil, Switzerland) until the start of hatching, at which point they were monitored daily.

A randomly selected subset of larvae from each treatment group (mean number ± SD: 49.4 ± 3.9) was photographed on the day of hatching with an Olympus C‐5060. Standard length of each larvae, as well as the length and width of their yolk sacs, was measured using the open‐access software IMAGEJ (http://rsb.info.nih.gov/ij/). Yolk sac volumes were calculated as described in Jensen et al. (2008).

### Treatment of embryos with silver nanoparticles

**Figure 1:**
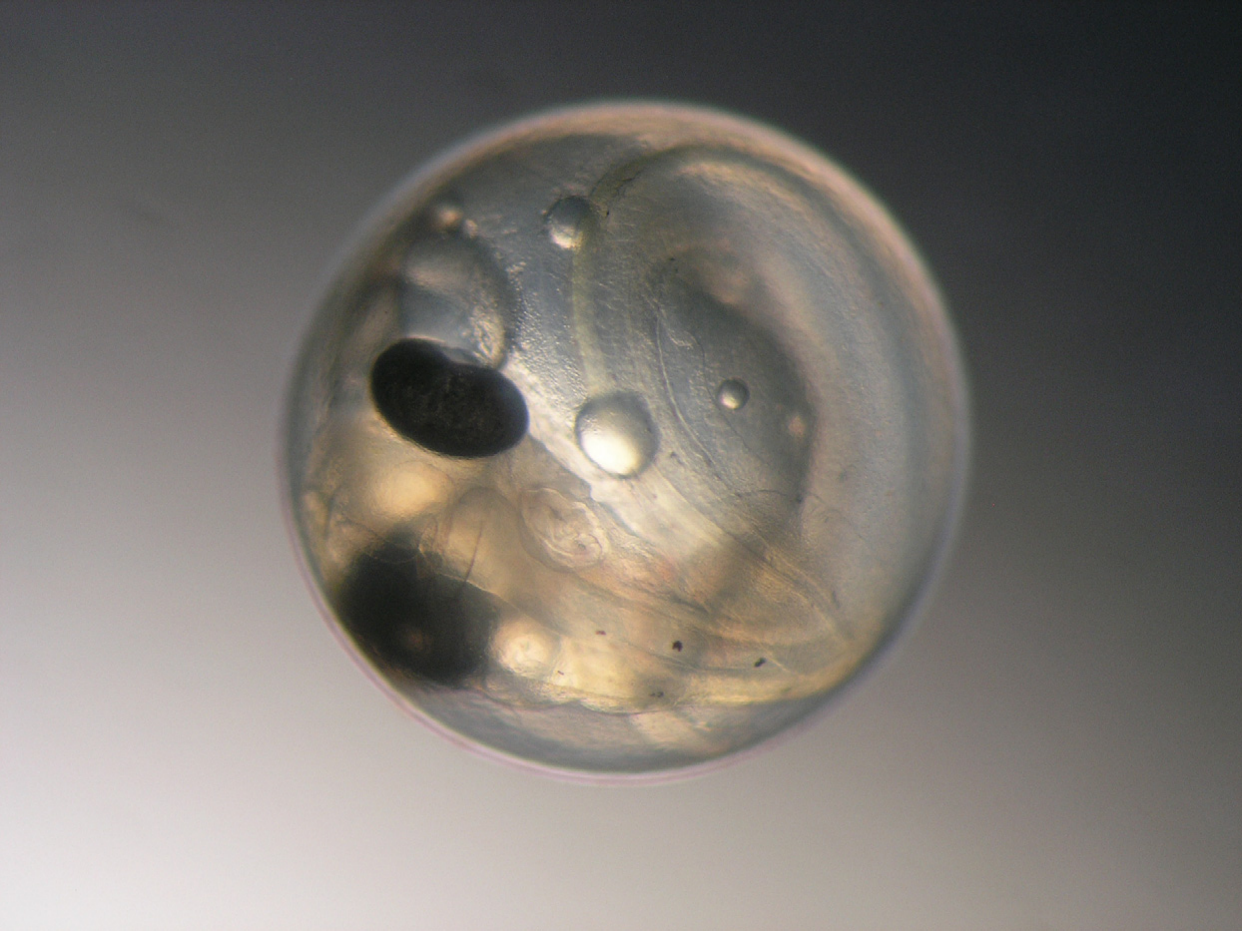
Whitefish embryo (*Coregonus palaea*). Photograph by M. Pompini.

At 240.5 DD postfertilization, embryos (Fig. 1) were divided into treatment groups (full factorial with regard to sibgroup). This time point was selected as it corresponds to the point in whitefish development in which maternal effects begin accounting for less variation in early life‐history traits, while additive genetic effects begin accounting for more (Clark et al. 2014). It was therefore deemed an appropriate time point to investigate heritable variation for silver tolerance. Each treatment included five to seven replicates per full‐sib family (depending on availability of embryos, *N*_total_ = 2896 singly raised embryos). Four subsets of embryos were assigned to the nanoparticle treatments and were treated with either 20 nm or 100 nm AgNP (Sigma‐Aldrich, St. Louis, MO) at concentrations of 0.5 *μ*g/L (low dose) or 100 *μ*g/L (high dose) per well. The low dose was chosen as it represents plausible baseline environmental concentrations. The high dose was selected as it approaches the upper limit of environmentally relevant concentrations, which is estimated to fall around 200 *μ*g/L (Odzak et al. 2015). All nanoparticles were diluted in standardized water and initially vortexed vigorously for 1 min, and then intermittently thereafter, to ensure that a homogenous solution was added to the wells. As the stock solutions of all AgNP treatments contained sodium citrate as a dispersant (2 mM), two groups of embryos also served as controls, receiving concentrations which corresponded to those present in either the low or high‐dose AgNP treatments, respectively, 5.2 × 10^−5^ mM and 0.01 mM per well. To determine whether equivalent amounts of silver in the ionic form would provoke similar phenotypes in embryos, two groups were also treated with silver nitrate (AgNO_3_, Sigma‐Aldrich), such that wells contained either 9.7 × 10^−12^ or 2 × 10^−9^ moles of silver per well, which matched the amount of silver in the low‐ and high‐dose nanoparticle treatments, respectively. A final group of embryos served as the untreated control and was sham‐treated with standardized water. After treatment, pH was monitored across experimental groups to assure no significant deviations from neutrality, and oxygen levels were monitored to verify that levels stayed above 4 mg/L.

### Statistical analyses

All analyses were performed in R (R Development Core Team 2011). Mortality was analyzed with generalized linear mixed models (GLMM) while time until hatching, larval length, and yolk sac volume were analyzed with linear mixed models (LMM) using the lme4 package (Bates et al. 2011). Yolk sac volume was log‐transformed prior to analysis. For the analyses of mortality and hatching time, treatment was entered as a fixed effect, while sire, dam, and sire × dam were entered as random effects. For the analyses of larval length and yolk sac volume, model structure was the same, except that time until hatching was added as an additional fixed effect. To examine the importance of the fixed effects on phenotype, log likelihood ratio tests (LRT) were used to determine whether incorporation of the variable significantly improved model fit in comparison with one lacking it. If the LRT indicated no significant improvement, we deferred to the model with fewer terms. If treatment was found to have a significant effect on phenotype, pairwise comparisons between groups were examined using the “glht function” in the “multcomp” package, which provides adjusted *P*‐values for multiple comparisons.

To assess the importance of each parental effect, a full model incorporating all random effects (sire, dam, and sire × dam) was first compared to a model lacking the sire × dam interaction effect. As the sire × dam effect was never found to improve model fit, the reference model was deemed to be one which included sire and dam effects. Then, sire models (i.e., models lacking the sire effect) and dam models (models lacking the dam effect) were compared to the reference model.

To examine the significance of interaction effects (e.g., sire × treatment) on phenotype, a random slope model that incorporated all random effects (sire, dam, and sire × dam) was compared to a model incorporating the interaction (random slope–intercept model). LRT were used as described above to compare model fits. For the aforementioned and subsequent analyses, the untreated and sodium citrate controls were grouped into a single control, as mortality within these controls was essentially null (one embryo died), and hatching time and larval lengths were comparable. In the analysis of interaction effects, the control was compared to each of the six silver treatments.

Variance components for the traits of interest were extracted from the mixed effect models and used to calculate the components of phenotypic variation in each treatment, as described in Lynch and Walsh for full‐factorial North Carolina II breeding designs (p. 509; 1998). Specifically, epistatic effects were assumed to be negligible, and additive genetic variance (*V*_A_) was calculated as 4× the sire component of variance. Dam variance (*V*_Dam_) included both maternal environmental and genetic effects. Dominance genetic effects were estimated as 4× the sire × dam effect. Residual variance (*V*_Res_) included environmental variance, in addition to ½ *V*_A_ and ¾ *V*_D_ (Kearsey and Pooni 1996). Narrow‐sense heritability was estimated by dividing *V*_A_ by total phenotypic variation, as described in Lynch and Walsh (1998).

As confidence intervals for the random effects are not calculated in the lme4 package, the MCMCglmm package (Hadfield 2010) was used to obtain 95% highest posterior density confidence intervals for all parental effects, as well as heritability estimates. For this Bayesian analysis, model structure was the same as in lme4, in that sire, dam, and sire × dam were entered as random effects. Models for each treatment were run over 6–7 million iterations, with a burn in of 100,000, and a thinning interval of 1000. Inverse‐gamma prior distributions (nu = 0.002, *V* = 1) were used for hatching time, larval length, and yolk sac volume. Traces were plotted to ensure that no pattern was apparent, and that values were widely spread. Heidelburger and Welch tests were run to check for convergence. In three of the 21 tests, a lack of convergence was found for the female effect (i.e., for length in low‐dose 20‐nm AgNP and for yolk sac volume in low‐dose 20‐nm and 100‐nm AgNP), although the traces passed visual inspection. The posterior modes of the different variance components were calculated and compared to the lme4 estimates (see Table S2). As some of estimates obtained from lme4 and MCMCglmm did not completely align, we calculated deviance information criterion (DIC) for each full model (including sire, dam, and sire × dam models), in addition to sire, dam, and sire × dam models, as described above for the lme4 analysis. Model fits were then compared, and we verified that the significance of the different terms (e.g., sire and dam effects) corresponded between the two analyses. A difference of DIC that was ≥10 indicated a significant difference in model fits (Woodward 2012).

To assess whether hatching time, larval length, and yolk sac volume could evolve independently in different environments, we tested for cross‐environmental trait correlations (Pearson's product moment correlation) using paternal sibgroup means. Using the family mean approach to test for genetic correlations provides a conservative assessment of whether the correlation is significantly different from 0, with the advantage that the correlation cannot exceed ±1 (Lynch and Walsh 1998). To correct for multiple testing, the alpha level was adjusted using a Bonferroni correction.

## Results

### Effects of treatment on offspring phenotypes

**Figure 2:**
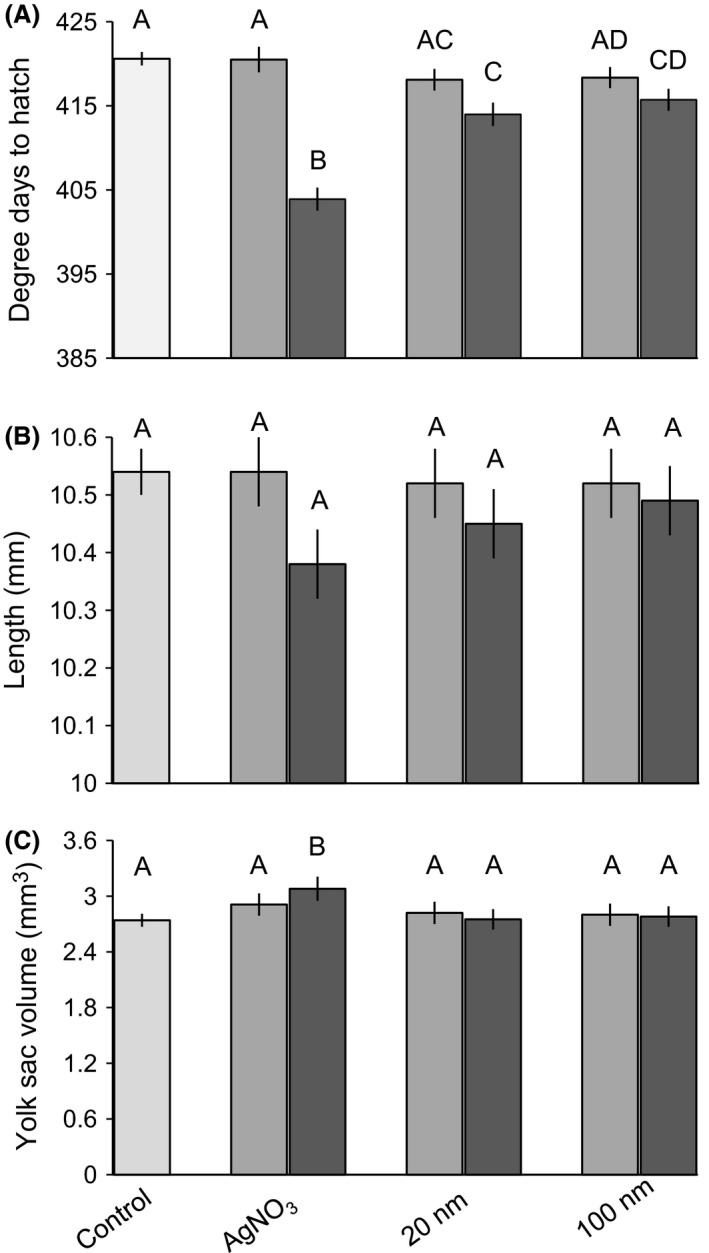
Mean degree days until hatching (A), larval length (B), and yolk sac volume (C) (± SE) in each treatment. Light gray bars correspond to the low‐dose (0.5 *μ*g/L) treatment, and dark gray to the high dose (100 *μ*g/L). Shared letters between bars denote no significant difference (See Results for statistics).

Treatment with ionic silver and AgNPs did not significantly impact embryonic survival in our experiment (LRT: *χ* ^2^ = 10.1, *P* = 0.12; in total 15 of 2896 embryos died, that is, overall mortality was 0.5%). In contrast, treatment had a marked impact on hatching time (LRT: *χ* ^2^ = 216.2, *P* < 0.001). In comparison with the control, embryos hatched significantly earlier in the high‐dose (100 *μ*g/L) AgNO_3_ treatment (*Z* = −14.1, *P* < 0.001), as well as in both high‐dose AgNP groups (20 nm: *Z* = −5.7, *P* < 0.001; 100 nm: *Z* = −4.2, *P* < 0.001) (Fig. 2A). Treatment with low‐dose (0.5 *μ*g/L) AgNO_3_ did not appear to induce hatching (*Z *= −0.2, *P* = 1.0). Similarly, embryos treated with the low doses of both sizes of AgNP did not hatch earlier than control embryos (20 nm: *Z* = −2.1, *P* = 0.33; 100 nm: *Z* = −2.0, *P* = 0.40).

The extent to which the different silver sources accelerated hatching was not the same, with both high‐dose AgNP treatments having a less pronounced effect than the AgNO_3_ group (20 nm: *Z* = 6.9, *P* < 0.001; 100 nm: *Z* = 8.1, *P* < 0.001). While ionic versus nanoparticulate silver generated different responses, there was no significant difference in hatching time between the 20 nm and 100 nm AgNP high‐dose treatments (*Z* = 1.2, *P* = 0.89).

Treatment was not found to significantly affect larval length (LRT: *χ* ^2^ = 2.5, *P* = 0.86; Fig. 2B); however, time until hatching did have a marked effect, with earlier hatching fish being smaller (LMM: *T* = 4.6, *P* < 0.001; Fig. 2B). Conversely, treatment had a significant effect on yolk sac volume (LRT: *χ* ^2^ = 14.2, *P* = 0.03), as did time until hatching (LMM: *T* = −3.8, *P* < 0.001) (Fig. 2C), with larvae from the high‐dose AgNO_3_ treatment having markedly larger yolk sacs (*T* = 3.2, *P* = 0.001).

### Genetic and maternal sources of variation on trait means and the reaction norms

**Figure 3:**
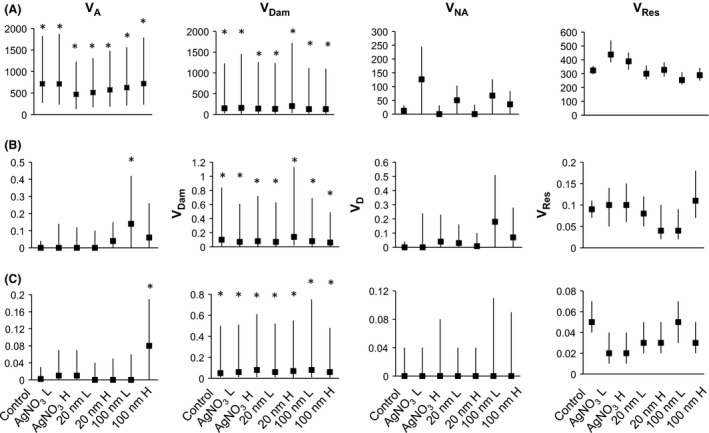
REML estimates of variance components (*V*_A_: additive genetic; *V*_D__am_: dam; *V*_NA_: nonadditive genetic; *V*_R__es_: residual) for hatching time (row A), larval length (B), and yolk sac volume (C) in each treatment (values provided in Table S2). Ninety‐five percent highest posterior density intervals are provided for each estimate. Asterisks indicate significant at *P* < 0.05, as determined by likelihood ratio tests (Tables S1, S4, and S5).

Additive genetic effects accounted for a significant part of variation in hatching time in every treatment (Fig. 3A, Tables S1–S3), and heritabilities were consistently high (always ≥ 0.7; Table S3). Similarly, dam effects on hatching time were significant across environments (Fig. 3A, Tables S1–S3). Dominance genetic effects had no significant effects on hatching time in either the control or any of the treatment groups (Fig. 3A, Tables S1–S3). Notably 95% HPD (highest posterior density) intervals were broad for all effects analyzed (Fig. 3, Table S3) for hatching time, as well as larval length and yolk sac volume (discussed below).

For larval length and yolk sac volume, dam effects were significant in every treatment (Fig. 3B and C, Tables S2–S5). In contrast, dominance genetic effects were never significant. Additive genetic effects on larval length were only significant in the low‐dose 100‐nm AgNP treatment (Fig. 3B, Table S4). Additive genetic effects on yolk sac volume were also significant only in one treatment, that is, the high‐dose 100‐nm AgNP. (Fig. 3C, Table S5).

While we found significant heritable variation for hatching time across treatments, we did not find any evidence of additive genetic variation for hatching plasticity (see nonsignificant t × s interactions in Table S6). We also found that hatching time was highly correlated across all experimental treatments (Table 1). Although there was no significant sire × treatment interaction, we found evidence of other interaction effects on hatching time: The three‐way interaction (sire × dam × treatment) was significant when comparing the control with the low‐dose AgNO_3_ treatment (LRT: *χ* ^2^ = 6.8, *P* = 0.03; Table S6), and the dam × treatment interaction was significant when comparing our control to the high‐dose 20‐nm AgNP group (LRT: *χ* ^2^ = 8.4, *P* = 0.01; Table S6).

**Table 1:** Genetic correlations (Pearson's correlation coefficients based on sire means) of hatching time (A), larval length (B), and yolk sac volume (C) across treatments

	Control	AgNO_3_	AgNO_3_	20 nm	20 nm	100 nm	100 nm
		L	H	L	H	L	H
(A) Hatching time							
Control	–	0.93^a^	0.97^a^	0.94^a^	0.95^a^	0.89^a^	0.97^a^
AgNO_3_ L	–	–	0.95^a^	0.86^a^	0.90^a^	0.92^a^	0.95^a^
AgNO_3_ H	–	–	–	0.93^a^	0.95^a^	0.87^a^	0.97^a^
20 nm L	–	–	–	–	0.96^a^	0.78^a^	0.95^a^
20 nm H	–	–	–	–	–	0.79^a^	0.95^a^
100 nm L	–	–	–	–	–	–	0.90^a^
100 nm H	–	–	–	–	–	–	–
(B) Larval length							
Control	–	0.17	0.29	0.35	0.41	0.55	0.21
AgNO_3_ L	–	–	−0.02	0.42	0.33	0.20	−0.30
AgNO_3_ H	–	–	–	−0.15	−0.01	0.06	−0.04
20 nm L	–	–	–	–	0.39	−0.03	0.23
20 nm H	–	–	–	–	–	0.37	0.26
100 nm L	–	–	–	–	–	–	0.21
100 nm H	–	–	–	–	–	–	–
(C) Yolk sac volume							
Control	–	0.44	−0.28	0.06	−0.52	0.37	0.52
AgNO_3_ L	–	–	0.05	0.02	−0.18	0.43	−0.01
AgNO_3_ H	–	–	–	0.48	0.65	0.19	0.25
20 nm L	–	–	–	–	0.24	0.33	0.35
20 nm H	–	–	–	–	–	−0.14	−0.33
100 nm L	–	–	–	–	–	–	0.37
100 nm H	–	–	–	–	–	–	–

a Significant after Bonferroni correction (*P* < 0.002).

With respect to larval length, we found one instance where the sire × treatment interaction term was significant, that is, when comparing the control to the low‐dose 100‐nm AgNP treatment (LRT: *χ* ^2^ = 6.0, *P* = 0.05; Table S7). No other interactions were significant (Table S7). Similarly, no interactions were found to have a significant effect on yolk sac volume (Table S8). For both traits, no significant correlations were found for sire means across any of the experimental treatments (Table 1).

## Discussion

We have described how prolonged exposure to low concentrations of both nanoparticulate and ionic silver affects early life‐history traits in a wild salmonid population. In the context of our experimental conditions, we found that treatment of whitefish embryos with both AgNP and silver nitrate did not increase embryonic mortality. In contrast, exposure during early developmental stages in zebrafish (*Danio rerio*) (Lee et al. 2007; Powers et al. 2010; Massarsky et al. 2013) and Japanese medaka (*Oryzias latipes*) (Kashiwada et al. 2012) has been linked to increased embryonic mortality. Notably, many of these studies exposed embryos to much higher concentrations of both silver nanoparticles and silver nitrate than the present study (though see Massarsky et al. 2013). Moreover, in these studies, embryos were generally challenged in the hours following fertilization, whereas we treated the whitefish embryos at the late‐eyed stage. Sensitivity to environmental stressors can change dramatically across development (Roleda et al. 2007; Rohr et al. 2010). Clark et al. (2014) demonstrated that susceptibility to pathogen challenge changed significantly over the course of embryonic development in the same population of whitefish. As tolerance to AgNP has been shown to change according to developmental stage in other fish species (Kashiwada et al. 2012; Cho et al. 2013), it is possible that such a dependency exists in whitefish.

Treatment with both silver sources also did not appear to directly affect growth during embryonic development. Instead, treatment with the higher dosages of AgNP and AgNO_3_ was associated with earlier hatching_,_ and hatching time ultimately affected length and yolk sac volume, that is, length increased with incubation time, while yolk sac volume decreased. Hatching, which is the first important niche switch for many species, is a plastic trait, and the timing of this event can be accelerated or delayed to cope with stage‐specific risks such as pathogens and predators in the environment in a wide range of taxa (e.g., Christy 2011; Doody 2011; Warkentin 2011; Pompini et al. 2013; Touchon and Wojdak 2014). Whitefish specifically have previously been shown to induce hatching to escape pathogens in their immediate environment (Wedekind 2002) or to avoid desiccation (Wedekind and Müller 2005). In the context of our experiment, it appears that exposure to higher concentrations of silver provoked a behavioral response in some embryos, in that they chose to hatch earlier, and potentially less developed than they otherwise would have under nonpolluted conditions. The fact that yolk sacs were significantly larger in the high‐dosage AgNO_3_ treatment, where mean hatching time was the earliest, is consistent with this. Notably, induced hatching in response to silver stress does not appear to be an evolutionarily conserved response, as other studies in zebrafish have found that exposure to silver ions and nanoparticles resulted in delayed hatching (Asharani et al. 2008; Bar‐Ilan 2009; Wu et al. 2010; Massarsky et al. 2013). Despite the delay in hatching, fish were also found to hatch at a smaller size, with reduced yolk sac volumes (Asharani et al. 2008; Bar‐Ilan 2009; Muth‐Köhne et al. 2013). These differences suggest that the effect that silver exposure has on an organism depends on the species at hand, and potentially, the point in development at which it is exposed.

In the absence of increased embryonic mortality, it is somewhat unexpected that embryos would hatch precociously and at a reduced size, as they would seemingly gain limited fitness benefits from the early niche switch. Although early emerging can be associated with positive fitness effects due to reduced predation (Brännäs 1995), smaller salmonid larvae generally have decreased swimming abilities and are consequently less able to escape predators and hunt (Einum and Fleming 2000; Jensen et al. 2008). This could place them at a distinct disadvantage in comparison with their larger, later‐emerging conspecifics. If early hatching is indeed associated with increased larval mortality under benign environmental conditions in this population of whitefish, the decision to hatch early would appear to be a maladaptive reaction to the silver cue. However, we cannot say categorically that the embryos were not in fact experiencing harmful effects of silver exposure. Silver nanoparticles have been observed to infiltrate cells and tissue of zebrafish embryos, as well as impair neurological development (Muth‐Köhne et al. 2013). It is therefore conceivable that events were taking place at the cellular and tissue level with sublethal effects that prompted embryos to make the life‐history switch, that is, to avoid a stressful environment. It is also possible that the whitefish accelerated hatching to mitigate future fitness consequences of exposure. For example, Powers et al. (2010) showed that treatment of zebrafish embryos with low concentrations (10 *μ*g/L) of soluble silver resulted in impaired swimming behavior, despite having not induced any gross changes in embryo phenotypes. Further studies are required to determine whether silver‐exposed whitefish embryos experience sublethal physiological deficits that decrease viability later in life. It would then be necessary to establish whether hatching earlier helps mitigate these effects in the face of silver pollution, directly resulting in higher survival at later developmental stages.

While both silver species induced hatching at the higher dosage, they did not do so to the same extent. Embryos hatched significantly faster in the silver nitrate treatment than in either AgNP group. No marked difference in hatching time was observed between groups that were exposed to differently sized nanoparticles. Previous studies have produced conflicting results concerning which silver species has a more pronounced impact on aquatic organisms, with some showing greater effects of free silver ions (e.g., Sakamoto et al. 2015) and others demonstrating stronger effects of nanoparticles (e.g., Griffitt et al. 2009). Whether the nanoparticles' elicited response stems from indirect (i.e., metal dissolution and release of silver ions) or direct effects (e.g., particle size, composition) is also a subject of debate (Navarro et al. 2008). In the context of our study, the effect that the AgNP had on the embryos appeared to be concentration dependent (i.e., only apparent at higher dosages), resulting potentially from the release of free silver, and not dependent on particle properties, such as size.

Although the higher concentration silver treatments did have a distinct effect on hatching time, they did not appear to have a pronounced effect on the amount of heritable variation for the trait, as additive genetic effects were significant across treatments. Notably, it is possible that treatment had more subtle effects on the amount of heritable variation that was responsible for phenotypic variation; however, the wide confidence intervals for additive genetic effects observed in the present study prevent us from drawing such conclusions. Further investigations utilizing more males may help obtain more precise estimates for the additive genetic component. Nevertheless, the fact that additive genetic effects remained significant in all environments suggest that silver exposure did not drastically decrease the evolutionary potential of the trait. In contrast, Gomez‐Mestre et al. found that induced hatching in a toad (*Bufo americanus*) (Gomez‐Mestre et al. 2008) and a treefrog (*Agalychnis callidryas*) (Gomez‐Mestre and Warkentin 2013) in response to pathogen and predator stressors, respectively, was characterized by reduced genetic variation, in comparison with spontaneous hatching. Cumulatively, these results suggest that the amount of heritable variation in induced versus spontaneous hatching likely depends on the species, how developmentally constrained it is in terms of early hatching, on the nature of the stressor, and on the strength of directional selection it has exerted on a population.

Notably, additive genetic effects on hatching time in our study were very large, resulting in heritabilities that sometimes exceeded one and even surpassed dam effects, which were also universally significant. Dam effects are typically expected to be larger than sire effects, as they encompass both maternal environmental and genetic effects; however, as only four dams were used in the experiment, more uncertainty is associated with these estimates, as indicated by the wide HPD intervals. These results may also be the consequence of epistatic genetic effects, which are assumed to be negligible in calculations of additive genetic variance (Lynch and Walsh 1998), but likely play an important role in determining embryo phenotype (Wedekind et al. 2008a).

While larval length and yolk sac volume were always strongly affected by maternal effects, our analyses also showed some evidence of environmental effects on additive genetic variation, that is, it accounted for a significant part of variation in the low‐ and high‐dose 100 nm treatments, respectively. This finding suggests that certain silver treatments provoked a release of cryptic genetic variation, but that the effect was dependent on the silver source and concentration. Stressful environmental conditions have previously been shown to provoke such a release of cryptic genetic variation (McGuigan and Sgro 2009); however, as these particular treatment groups did not differ in mean phenotype with the control, it is not immediately obvious that they were affected by exposure. Our results suggest that a comprehensive analysis not only of phenotypic means, but also of the quantitative genetic architecture of traits aid in understanding the effects of ecological changes on wild populations.

As with trait means, the strength of interaction effects on phenotype depended on the trait and treatment in question. In the case of hatching time, we found evidence of treatment × dam effects in the high‐dose 20‐nm AgNP group, which indicates that progeny reacted differently according to maternal genetic and/or environmental contributions. We also observed treatment × sire × dam effects in the low concentration silver nitrate group, which suggests an interaction between certain genotypes and treatment. However, we found no indications of additive genetic variation for hatching reaction norms. Hatching time was even genetically correlated across treatments. Cross‐environmental trait correlations indicate that a trait is under the same genetic control in different environments, which can ultimately constrain the evolution of reaction norms (Via 1984). Interestingly, another study conducted on the same population of whitefish found no evidence of genetic variation for hatching plasticity following pathogen challenge (Clark et al. 2013b). Together, these results suggest an overall limitation on the evolution of hatching reaction norms in these whitefish.

Unlike hatching time, our analysis of larval length reaction norms did reveal heritable genetic variation in one instance, that is, when comparing the control to the low‐dose 100‐nm treatment. The fact that this is the same treatment wherein we observed a release of heritable variation suggests an interaction between certain alleles and the specific treatment.

The reaction norm of this trait does therefore appear to have the genetic variation necessary for evolution. Moreover, length, such as yolk sac volume, was never found to be genetically correlated across environments, indicating less of a constraint on the evolution of plasticity.

In conclusion, exposure of whitefish embryos to certain concentrations of AgNP and AgNO_3_ did not result in increased mortality or directly decrease growth during development, but did spur a behavioral response, in that some embryos decided to hatch earlier, and therefore at a smaller size. To determine whether this is an adaptive response, allowing embryos to mitigate potential sublethal physiological effects of silver, or rather a maladaptive response to an otherwise benign chemical cue, it would first be necessary to establish whether low‐level silver exposure during embryonic development is indeed associated with decreased fitness at later life stages. If this were the case, one would then need to determine whether early hatching is directly linked to increased survivorship at later developmental time points. Exploring these research avenues further is of particular importance, in light of the fact that no additive genetic variation was found for hatching plasticity. The hatching reaction norm therefore has limited potential to rapidly evolve, and whitefish progeny will likely continue reacting more or less uniformly to the silver stimulus. At the same time, our results provided some indications that silver exposure could provoke a release of heritable variation, but that this appears highly context dependent (i.e., dependent on trait and silver source, and concentration of silver).

Our study provides the first experimental analyses on the potential consequences of low‐level silver exposure on early life stages of a wild population. Such studies are currently lacking, but are indeed critical as silver from engineered nanoparticles is expected to increase drastically in concentrations in the aquatic environment (Handy et al. 2008). While we know that these particles can prove lethal to model organisms, we must better understand how they affect members stemming from wild populations, in terms of both the phenotype and the quantitative genetic architecture of fitness traits. Such understanding is necessary to anticipate how silver particles will affect species at the population level and help determine what are environmentally acceptable concentrations.

## Data Accessibility

Data for this study are deposited in the Dryad Digital Repository (doi: 10.5061/dryad.dv4rg).

## Supporting information


**Table S1.** Mixed model linear regression on time until hatching within each treatment.
**Table S2.** Comparison of deviance information criterion (DIC) of the full model (with all random effects) to the reference model (in bold), to sire and dam models, as estimated with MCMCglmm.
**Table S3.** Variance component (VA: additive genetic; VDam: dam; VNA: nonadditive genetic; VRes: residual) and heritability estimates for each trait and treatment, and 95% HPD intervals.
**Table S4.** Mixed model linear regression on larval length within each treatment.
**Table S5.** Mixed model linear regression on yolk sac volume within treatments.
**Table S6.** Likelihood ratio tests on mixed model logistic regressions on hatching time.
**Table S7.** Likelihood ratio tests on mixed model logistic regressions on larval length.
**Table S8.** Likelihood ratio tests on mixed model logistic regressions on yolk sac volume.
