# Supplementary material for: Genetic correlations and little genetic variance for reaction norms may limit potential for adaptation to pollution by ionic and nanoparticulate silver in a whitefish (Salmonidae)
Source: Ecol Evol. 2016 Mar 17;6(9):2751–62. doi: 10.1002/ece3.2088 (PMC4798832; doi:10.1002/ece3.2088)
Supplement: Supplementary file 1 — Table S1. Mixed model linear regression on time until hatching within each treatment. Table S2. Comparison of deviance information criterion (DIC) of the full model (with all random effects) to the reference model (in bold), to sire and dam models, as estimated with MCMCglmm. Table S3. Variance component (VA: additive genetic; VDam: dam; VNA: nonadditive genetic; VRes: residual) and heritability estimates for each trait and treatment, and 95% HPD intervals. Table S4. Mixed model linear regression on larval length within each treatment. Table S5. Mixed model linear regression on yolk sac volume within treatments. Table S6. Likelihood ratio tests on mixed model logistic regressions on hatching time. Table S7. Likelihood ratio tests on mixed model logistic regressions on larval length. Table S8. Likelihood ratio tests on mixed model logistic regressions on yolk sac volume. [file ECE3-6-2751-s001.docx]

***Supplementary material: Tables S1 – S8***

**Genetic correlations and little genetic variance for reaction norms may limit potential for adaptation to pollution by ionic and nanoparticulate silver in a whitefish (Salmonidae)**

Emily S. Clark, Manuel Pompini, Anshu Uppal, and Claus Wedekind

Department of Ecology and Evolution, Biophore, University of Lausanne, 1015, Lausanne, Switzerland

Table S1. Mixed model linear regression on time until hatching within each treatment.

| Model | Effect Tested | AIC | χ^2^ | P |
| --- | --- | --- | --- | --- |
| *Control* |  |  |  |  |
| s + d + sxd |  | 8278.1 |  |  |
| **s + d** | s x d | 8276.6 | 0.5 | 0.48 |
| d | s | 8617.9 | 343.3 | <0.001 |
| s | d | 8550.0 | 275.5 | <0.001 |
| *AgNO_3_ low* |  |  |  |  |
| s + d + sxd |  | 2944.9 |  |  |
| **s + d** | s x d | 2945.4 | 2.6 | 0.11 |
| d | s | 3015.1 | 71.6 | <0.001 |
| s | d | 3008.0 | 64.6 | <0.001 |
| *AgNO_3_ high* |  |  |  |  |
| s + d + sxd |  | 2863.3 |  |  |
| **s + d** | s x d | 2861.3 | 0 | 1 |
| d | s | 2911.3 | 52.0 | <0.001 |
| s | d | 2924.0 | 64.8 | <0.001 |
| *20 nm low* |  |  |  |  |
| s + d + sxd |  | 2810.2 |  |  |
| **s + d** | s x d | 2809.2 | 1.0 | 0.31 |
| d | s | 2884.3 | 77.1 | <0.001 |
| s | d | 2886.5 | 79.3 | <0.001 |
| *20 nm high* |  |  |  |  |
| s + d + sxd |  | 2805.2 |  |  |
| **s + d** | s x d | 2803.2 | 0 | 1 |
| d | s | 2878.9 | 77.7 | <0.001 |
| s | d | 2914.2 | 113.1 | <0.001 |
| *100 nm low* |  |  |  |  |
| s + d + sxd |  | 2773.1 |  |  |
| **s + d** | s x d | 2773.2 | 2.2 | 0.14 |
| d | s | 2879.1 | 107.8 | <0.001 |
| s | d | 2860.8 | 89.5 | <0.001 |
| *100 nm high* |  |  |  |  |
| s + d + sxd |  | 2800.0 |  |  |
| **s + d** | s x d | 2798.4 | 0.5 | 0.49 |
| d | s | 2905.6 | 109.2 | <0.001 |
| s | d | 2875.1 | 78.6 | <0.001 |

Sire (s), dam (d), and sire x dam (sxd) were entered as random effects. The significance of the sire x dam interaction effect was first examined by comparing the full model (incorporating all random effects) to a model lacking it. Since the interaction was never significant, all further models were compared to the reference model (in bold). Likelihood ratio tests (LRT) were used to compare model fits (χ^2^). Akaike’s information criteria (AIC), which provide a measure of model fit and model complexity (lower values indicate a better fit to the data) are also provided.

Table S2. Comparison of deviance information criterion (DIC) of the full model (with all random effects) to the reference model (in bold), to sire and dam models, as estimated with MCMCglmm. As with Akaike’s information criterion (AIC), the lower value indicates the better model fit. Models were run 6-7 million iterations, with a burn in of 100,000, and a thinning interval of 1000. Inverse-Gamma prior distributions (nu= 0.002, V = 1) were used.

| Model | Effect Tested | DIC | | |
| --- | --- | --- | --- | --- |
|  |  | Hatching time | Larval length | Yolk sac volume |
| *Control* |  |  |  |  |
| s + d + sxd |  | 8225.8 | 69.4 | 1.6 |
| **s + d** | s x d | 8225.6 | 67.3 | -0.2 |
| d | s | **8602.0** | 65.3 | -1.5 |
| s | d | **8517.0** | **144.5** | **74.6** |
| *AgNO_3_ low* |  |  |  |  |
| s + d + sxd |  | 2913.0 | 32.9 | -30.3 |
| **s + d** | s x d | 2914.2 | 34.7 | -32.4 |
| d | s | **3004.1** | 33.8 | -31.1 |
| s | d | **2990.0** | **52.3** | **11.7** |
| *AgNO_3_ high* |  |  |  |  |
| s + d + sxd |  | 2833.1 | 40.8 | -30.7 |
| **s + d** | s x d | 2832.6 | 42.1 | -28.4 |
| d | s | **2899.9** | 41.4 | -27.0 |
| s | d | **2909.0** | **62.0** | **24.9** |
| *20 nm low* |  |  |  |  |
| s + d + sxd |  | 2776.4 | 33.0 | -22.7 |
| **s + d** | s x d | 2776.5 | 33.9 | -23.5 |
| d | s | **2872.5** | 32.4 | -25.0 |
| s | d | **2868.1** | **55.7** | **24.0** |
| *20 nm high* |  |  |  |  |
| s + d + sxd |  | 2769.3 | -2.6 | -16.6 |
| **s + d** | s x d | 2768.8 | -1.6 | -17.9 |
| d | s | **2865.6** | 2.0 | -19.4 |
| s | d | **2897.1** | **42.9** | **17.9** |
| *100 nm low* |  |  |  |  |
| s + d + sxd |  | 2735.7 | 10.2 | 1.5 |
| **s + d** | s x d | 2736.7 | 20.1 | 2.1 |
| d | s | **2867.4** | **35.2** | -0.1 |
| s | d | **2839.4** | **52.3** | **34.8** |
| *100 nm high* |  |  |  |  |
| s + d + sxd |  | 2762.1 | 48.1 | -19.8 |
| **s + d** | s x d | 2762.1 | 49.6 | -20.2 |
| d | s | **2894.4** | 51.8 | *-10.2* |
| s | d | **2853.0** | **61.0** | **21.5** |

Bold values indicate a difference in DIC between the reference model and the sire and dam models of at least 10. Italicized values indicate a ΔDIC which approaches 10.

Table S3. Variance component (V_A_: additive genetic; V_Dam_: dam; V_NA_: nonadditive genetic; V_Res_: residual) and heritability estimates for each trait and treatment, and 95% HPD intervals.

| Treatment | V_A_ | V_Dam_ | V_NA_ | V_Res_ | *h*^2^ |
| --- | --- | --- | --- | --- | --- |
| *A) Hatching time* | |  |  |  |  |
| Control | **713.2**; 579.9 | **148.4**; 97.5 | **12.2**; 0.06 | **323.2**; 322.2 | **1.1**; 0.94 |
|  | [269.1, 1822.6] | [26.3, 1228.8] | [0, 31.4] | [296.7, 355.7] | [0.13, 1.94] |
| AgNO_3_ low | **711.2**; 569.5 | **157.0**; 138.2 | **126.4**; 0.39 | **438.1**; 445.6 | **0.8**; 0.76 |
|  | [229.9 , 1870.3] | [16.1, 1453.2] | [0, 244.8] | [381.9, 540.0] | 0.12, 1.68] |
| AgNO3 high | **468.8**; 376.5 | **140.7**; 104.7 | **0**; 0.37 | **388.8**; 400.0 | **0.7**; 0.60 |
|  | [125.7, 1227.2] | [19.7, 1258.9] | [0, 31.7] | [328.1, 452.3] | [0.07, 1.44] |
| 20 nm low | **510.4**; 459.6 | **133.2**; 76.3 | **50.4**; 0.02 | **300.1**; 309.0 | **0.9**; 0.83 |
|  | [170.0, 1313.7] | [17.0, 1236.4] | [0, 103.1] | [258.4, 358.0] | [0.13, 1.72] |
| 20 nm high | **572.0**; 410.5 | **201.4**; 210.4 | **0**; 0.22 | **327.1**; 320.8 | **0.8**; 0.63 |
|  | [182.9, 1479.5] | [29.4, 1722.2] | [0, 34.4] | [278.7, 382.4] | [0.05, 1.61] |
| 100 nm low | **625.2**; 519.3 | **128.5**; 106.3 | **67.2** ; 0.12 | **253.8**; 256.7 | **1.1**; 0.90 |
|  | [210.7, 1565.2] | [15.7, 1115.9] | [0, 126.4] | [221.7, 309.9] | [0.18, 2.00] |
| 100nm high | **718.4**; 620.7 | **128.7**; 125.9 | **35.6**; 0.15 | **289.0**; 293.7 | **1.2**; 1.0 |
|  | [230.1, 1787.3] | [13.2, 1099.4] | [0, 83.3] | [249.7, 342.1] | [0.20, 2.09] |
|  |  |  |  |  |  |
| *B) Length* |  |  |  |  |  |
| Control | **0;** 0.004 | **0.10;** 0.12 | **0;** 0.004 | **0.09**; 0.08 | **0**; 0.01 |
|  | [0.001, 0.04] | [0.01, 0.84] | [0.001, 0.04] | [0.07, 0.11] | [0.001, 0.20] |
| AgNO_3_ low | **0**; 0.004 | **0.07**; 0.03 | **0**; 0.006 | **0.10**; 0.08 | **0**; 0.02 |
|  | [0.001, 0.14] | [0.002, 0.61] | [0.002, 0.24] | [0.05, 0.14] | [0.001, 0.71] |
| AgNO3 high | **0**; 0.005 | **0.08**; 0.05 | **0.04**; 0.01 | **0.10**; 0.10 | **0**; 0.01 |
|  | [0.001, 0.12] | [0.0005, 0.72] | [0.001, 0.23] | [0.06, 0.15] | [0.0002, 0.50] |
| 20 nm low | **0**; 0.004 | **0.07**; 0.12 | **0.03**; 0.004 | **0.08**; 0.07 | **0**; 0.02 |
|  | [0.001, 0.10] | [0.001, 0.63] | [0.001, 0.16] | [0.05, 0.12] | [0.0001, 0.51] |
| 20 nm high | **0.04**; 0.01 | **0.14**; 0.12 | **0.008**; 0.004 | **0.04**; 0.04 | **0.2**; 0.02 |
|  | [0.001, 0.15] | [0.02, 1.13] | [0.001, 0.10] | [0.02, 0.07] | [0.0001, 0.67] |
| 100 nm low | **0.14**; 0.01 | **0.08**; 0.06 | **0.18**; 0.01 | **0.04**; 0.04 | **0.7**; 0.02 |
|  | [0.001, 0.42] | [0.0003, 0.70] | [0.001, 0.51] | [0.02, 0.09] | [0.001, 1.56] |
| 100nm high | **0.06**; 0.01 | **0.06**; 0.05 | **0.07**; 0.01 | **0.11**; 0.11 | **0.3**; 0.02 |
|  | [0.001, 0.26] | [0.0003, 0.49] | [0.001, 0.28] | [0.07, 0.18] | [0.0002, 1.02] |
|  |  |  |  |  |  |
| *C) Yolk sac volume* | |  |  |  |  |
| Control | **0.002;** 0.003 | **0.05;** 0.05 | **0;** 0.003 | **0.05**; 0.05 | **0.02**; 0.02 |
|  | [0.001, 0.03] | [0.01, 0.50] | [0.001, 0.03] | [0.04, 0.07] | [0.0002, 0.25] |
| AgNO_3_ low | **0.01**; 0.01 | **0.06**; 0.05 | **0**; 0.003 | **0.02**; 0.02 | **0.1**; 0.02 |
|  | [0.001, 0.07] | [0.01, 0.51] | [0.001, 0.03] | [0.01, 0.04] | [0.0005, 0.70] |
| AgNO3 high | **0.01**; 0.005 | **0.08**; 0.06 | **0**; 0.003 | **0.02**; 0.02 | **0.1**; 0.03 |
|  | [0.001, 0.07] | [0.01, 0.61] | [0.001, 0.08] | [0.01, 0.04] | [0.0002, 0.63] |
| 20 nm low | **0**; 0.004 | **0.06**; 0.002 | **0**; 0.004 | **0.03**; 0.03 | **0**; 0.02 |
|  | [0.001, 0.04] | [0.01, 0.52] | [0.001, 0.04] | [0.02, 0.05] | [0, 0.43] |
| 20 nm high | **0**; 0.003 | **0.07**; 0.07 | **0**; 0.003 | **0.03**; 0.03 | **0**; 0.02 |
|  | [0.003, 0.05] | [0.005, 0.55] | [0.001, 0.04] | [0.02, 0.05] | [0.0003, 0.45] |
| 100 nm low | **0**; 0.004 | **0.08**; 0.20 | **0**; 0.003 | **0.05**; 0.05 | **0**; 0.01 |
|  | [0.001, 0.06] | [0.009, 0.75] | [0.001, 0.11] | [0.03, 0.07] | [0.002, 0.41] |
| 100nm high | **0.08**; 0.01 | **0.06**; 0.05 | **0**; 0.003 | **0.03**; 0.02 | **0.7**; 0.1 |
|  | [0.001, 0.19] | [0.006, 0.48] | [0.001, 0.07] | [0.02 0.05] | [0.001, 1.34] |

REML estimates extracted from full models (see Tables S1, S3-4) for each variance component are in bold, followed by the estimates obtained from MCMCglmm. Numbers in brackets indicate the 95% HPD interval for each estimate.

Table S4. Mixed model linear regression on larval length within each treatment.

| Model | Effect Tested | AIC | χ^2^ | P |
| --- | --- | --- | --- | --- |
| *Control* |  |  |  |  |
| Time to hatch + s + d + sxd |  | 83.1 |  |  |
| **Time to hatch +** **s + d** | s x d | 81.2 | 0 | 1 |
| Time to hatch + d | s | 79.2 | 0 | 1 |
| Time to hatch + s | d | 146.8 | 67.6 | <0.001 |
| *AgNO_3_ low* |  |  |  |  |
| Time to hatch + s + d + sxd |  | 45.3 |  |  |
| **Time to hatch +** **s + d** | s x d | 43.2 | 0 | 1 |
| Time to hatch + d | s | 41.2 | 0 | 1 |
| Time to hatch + s | d | 53.2 | 11.9 | <0.001 |
| *AgNO_3_ high* |  |  |  |  |
| Time to hatch + s + d + sxd |  | 52.8 |  |  |
| **Time to hatch +** **s + d** | s x d | 51.2 | 0.4 | 0.53 |
| Time to hatch + d | s | 49.2 | 0 | 1 |
| Time to hatch + s | d | 62.5 | 13.3 | <0.001 |
| *20 nm low* |  |  |  |  |
| Time to hatch + s + d + sxd |  | 45.1 |  |  |
| **Time to hatch +** **s + d** | s x d | 43.3 | 0.2 | 0.64 |
| Time to hatch + d | s | 41.3 | 0 | 1 |
| Time to hatch + s | d | 58.3 | 17.0 | <0.001 |
| *20 nm high* |  |  |  |  |
| Time to hatch + s + d + sxd |  | 16.6 |  |  |
| **Time to hatch +** **s + d** | s x d | 14.6 | 0.02 | 0.86 |
| Time to hatch + d | s | 14.0 | 1.4 | 0.24 |
| Time to hatch + s | d | 43.5 | 30.1 | <0.001 |
| *100 nm low* |  |  |  |  |
| Time to hatch + s + d + sxd |  | 38.0 |  |  |
| **Time to hatch +** **s + d** | s x d | 39.4 | 3.4 | 0.06 |
| Time to hatch + d | s | 43.8 | 6.4 | 0.01 |
| Time to hatch + s | d | 55.7 | 18.3 | <0.001 |
| *100 nm high* |  |  |  |  |
| Time to hatch + s + d + sxd |  | 59.7 |  |  |
| **Time to hatch +** **s + d** | s x d | 58.2 | 0.5 | 0.49 |
| Time to hatch + d | s | 57.9 | 1.8 | 0.18 |
| Time to hatch + s | d | 63.8 | 7.6 | 0.006 |

Time until hatching (degree days) was entered as a fixed effect, while sire (s), dam (d), and sire x dam (s x d) were entered as random effects within each model. Model selection was performed as described for Table S1.

Table S5. Mixed model linear regression on yolk sac volume within treatments.

| Model | Effect Tested | AIC | χ^2^ | P |
| --- | --- | --- | --- | --- |
| *Control* |  |  |  |  |
| Time to hatch + s + d + sxd |  | 15.5 |  |  |
| **Time to hatch +** **s + d** | s x d | 13.5 | 0 | 1 |
| Time to hatch + d | s | 11.6 | 0.1 | 0.83 |
| Time to hatch + s | d | 75.7 | 64.0 | <0.001 |
| *AgNO_3_ low* |  |  |  |  |
| Time to hatch + s + d + sxd |  | -15.6 |  |  |
| **Time to hatch +** **s + d** | s x d | -17.6 | 0 | 1 |
| Time to hatch + d | s | -19.3 | 0.3 | 0.59 |
| Time to hatch + s | d | 12.1 | 31.7 | <0.001 |
| *AgNO_3_ high* |  |  |  |  |
| Time to hatch + s + d + sxd |  | -10.9 |  |  |
| **Time to hatch +** **s + d** | s x d | -12.9 | 0 | 1 |
| Time to hatch + d | s | -14.1 | 0.7 | 0.39 |
| Time to hatch + s | d | 26.7 | 41.6 | <0.001 |
| *20 nm low* |  |  |  |  |
| Time to hatch + s + d + sxd |  | -8.9 |  |  |
| **Time to hatch +** **s + d** | s x d | -10.9 | 0 | 1 |
| Time to hatch + d | s | -12.9 | 0 | 1 |
| Time to hatch + s | d | 26.1 | 38.9 | <0.001 |
| *20 nm high* |  |  |  |  |
| Time to hatch + s + d + sxd |  | -4.0 |  |  |
| **Time to hatch +** **s + d** | s x d | -6.0 | 0 | 1 |
| Time to hatch + d | s | -8.0 | 0 | 1 |
| Time to hatch + s | d | 18.4 | 26.4 | <0.001 |
| *100 nm low* |  |  |  |  |
| Time to hatch + s + d + sxd |  | 14.7 |  |  |
| **Time to hatch +** **s + d** | s x d | 12.7 | 0 | 1 |
| Time to hatch + d | s | 10.7 | 0 | 1 |
| Time to hatch + s | d | 35.2 | 24.5 | <0.001 |
| *100 nm high* |  |  |  |  |
| Time to hatch + s + d + sxd |  | -0.9 |  |  |
| **Time to hatch +** **s + d** | s x d | -2.9 | 0 | 1 |
| Time to hatch + d | s | -0.2 | 4.8 | 0.03 |
| Time to hatch + s | d | 24.5 | 29.4 | <0.001 |

Time until hatching (degree days) was entered as a fixed effect, and parental origins were entered as random effects within each model. Model selection was performed as in Tables S1 and S3.

Table S6. Likelihood ratio tests on mixed model logistic regressions on hatching time.

| Model | Effect Tested | AIC | χ ^2^ | P |
| --- | --- | --- | --- | --- |
| *A) Control vs. AgNO_3_ low* | |  |  |  |
| **t + s + d + sxd** |  | 11196 |  |  |
| t + t\|s + d + sxd | t x s | 11200 | 0.95 | 0.62 |
| t + s + t\|d + sxd | t x d | 11200 | 0.12 | 0.94 |
| t + s + d + t\|sxd | t x s x d | 11194 | 6.82 | 0.03 |
| *B) Control vs. AgNO_3_ high* | |  |  |  |
| **t + s + d + sxd** |  | 11106 |  |  |
| t + t\|s + d + sxd | t x s | 11107 | 3.20 | 0.20 |
| t + s + t\|d + sxd | t x d | 11107 | 3.20 | 0.20 |
| t + s + d + t\|sxd | t x s x d | 11111 | 0 | 1 |
| *C) Control vs. 20 nm low* | |  |  |  |
| **t + s + d + sxd** |  | 11047 |  |  |
| t + t\|s + d + sxd | t x s | 11049 | 2.44 | 0.30 |
| t + s + t\|d + sxd | t x d | 11051 | 0.35 | 0.84 |
| t + s + d + t\|sxd | t x s x d | 11051 | 0.57 | 0.75 |
| *D) Control vs. 20 nm high* | |  |  |  |
| **t + s + d + sxd** |  | 11052 |  |  |
| t + t\|s + d + sxd | t x s | 11054 | 1.22 | 0.54 |
| t + s + d + sxd | t x d | 11047 | 8.40 | 0.01 |
| t + s + d + t\|sxd | t x s x d | 11053 | 2.31 | 0.32 |
| *E) Control vs. 100 nm low* | |  |  |  |
| **t + s + d + sxd** |  | 11028 |  |  |
| t + t\|s + d + sxd | t x s | 11027 | 5.65 | 0.06 |
| t + s + t\|d + sxd | t x d | 11032 | 0.49 | 0.78 |
| t + s + d + t\|sxd | t x s x d | 11027 | 5.36 | 0.07 |
| *F) Control vs. 100 nm high* | |  |  |  |
| **t + s + d + sxd** |  | 11033 |  |  |
| t + t\|s + d + sxd | t x s | 11037 | 0 | 1 |
| t + s + d + sxd | t x d | 11034 | 2.30 | 0.32 |
| t + s + d + t\|sxd | t x s x d | 11036 | 0.08 | 0.96 |

Treatment (t) was entered as a fixed effect (two levels including control and one of the silver groups), while sire (s), dam (d), and sire x dam (sxd) were entered as random effects. To test for an interaction between treatment and a random effect, a model incorporating the interaction term (random slope-intercept model) was compared to the reference model (random slope model, in bold). Akaike’s information criteria (AIC) provide a measure of model fit and model complexity (lower values indicate a better fit to the data). Likelihood ratio tests (LRT) were used to compare model fits (χ^2^).

Table S7. Likelihood ratio tests on mixed model logistic regressions on larval length.

| Model | Effect Tested | AIC | χ ^2^ | P |
| --- | --- | --- | --- | --- |
| *A) Control vs. AgNO_3_ low* | |  |  |  |
| **t + ht + s + d + sxd** |  | 108.3 |  |  |
| t + ht **+** t\|s + d + sxd | t x s | 112.2 | 0.04 | 0.98 |
| t + ht + s + t\|d + sxd | t x d | 111.8 | 0.48 | 0.79 |
| t + ht **+** s + d + t\|sxd | t x s x d | 112.3 | 0 | 1 |
| *B) Control vs. AgNO_3_ high* | |  |  |  |
| **t + ht + s + d + sxd** |  | 119.3 |  |  |
| t + ht **+** t\|s + d + sxd | t x s | 123.3 | 0 | 1 |
| t + ht + s + t\|d + sxd | t x d | 123.3 | 0 | 1 |
| t + ht **+** s + d + t\|sxd | t x s x d | 122.2 | 1.1 | 0.57 |
| *C) Control vs. 20 nm low* | |  |  |  |
| **t + ht + s + d + sxd** |  | 111.9 |  |  |
| t + ht **+** t\|s + d + sxd | t x s | 115.9 | 0 | 1 |
| t + ht + s + t\|d + sxd | t x d | 115.9 | 0 | 1 |
| t + ht **+** s + d + t\|sxd | t x s x d | 115.9 | 0 | 1 |
| *D) Control vs. 20 nm high* | |  |  |  |
| **t + ht + s + d + sxd** |  | 86.3 |  |  |
| t + ht **+** t\|s + d + sxd | t x s | 89.8 | 0.5 | 0.80 |
| t + ht + s + t\|d + sxd | t x d | 90.1 | 0.2 | 0.92 |
| t + ht **+** s + d + t\|sxd | t x s x d | 90.2 | 0 | 1 |
| *E) Control vs. 100 nm low* | |  |  |  |
| **t + ht + s + d + sxd** |  | 107.4 |  |  |
| t + ht **+** t\|s + d + sxd | t x s | 105.4 | 6.0 | **0.05** |
| t + ht + s + t\|d + sxd | t x d | 111.4 | 0.04 | 0.97 |
| t + ht **+** s + d + t\|sxd | t x s x d | 110.3 | 1.1 | 0.57 |
| *F) Control vs. 100 nm high* | |  |  |  |
| **t + ht + s + d + sxd** |  | 130.4 |  |  |
| t + ht **+** t\|s + d + sxd | t x s | 130.6 | 3.9 | 0.14 |
| t + ht + s + t\|d + sxd | t x d | 133.5 | 1.0 | 0.61 |
| t + ht **+** s + d + t\|sxd | t x s x d | 130.1 | 4.4 | 0.11 |

Treatment and degree days until hatching (ht) were entered as fix effects, while parental origin was entered as random effects. Model fit was assessed as in Table S6.

Table S8. Likelihood ratio tests on mixed model logistic regressions on yolk sac volume.

| Model | Effect Tested | AIC | χ^2^ | P |
| --- | --- | --- | --- | --- |
| *A) Control vs. AgNO_3_ low* | |  |  |  |
| **t + ht + s + d + sxd** |  | -8.5 |  |  |
| t + ht **+** t\|s + d + sxd | t x s | -4.6 | 0.07 | 0.97 |
| t + ht + s + t\|d + sxd | t x d | -4.5 | 0 | 1 |
| t + ht **+** s + d + t\|sxd | t x s x d | -4.5 | 0 | 1 |
| *B) Control vs. AgNO_3_ high* | |  |  |  |
| **t + ht + s + d + sxd** |  | -9.1 |  |  |
| t + ht **+** t\|s + d + sxd | t x s | -5.1 | 0 | 1 |
| t + ht + s + t\|d + sxd | t x d | -5.9 | 0.85 | 0.65 |
| t + ht **+** s + d + t\|sxd | t x s x d | -5.2 | 0.03 | 0.99 |
| *C) Control vs. 20 nm low* | |  |  |  |
| **t + ht + s + d + sxd** |  | -10.4 |  |  |
| t + ht **+** t\|s + d + sxd | t x s | -6.6 | 0.17 | 0.92 |
| t + ht + s + t\|d + sxd | t x d | -6.7 | 0.29 | 0.86 |
| t + ht **+** s + d + t\|sxd | t x s x d | -6.5 | 0.01 | 0.99 |
| *D) Control vs. 20 nm high* | |  |  |  |
| **t + ht + s + d + sxd** |  | -5.5 |  |  |
| t + ht **+** t\|s + d + sxd | t x s | -2.6 | 1.0 | 0.59 |
| t + ht + s + t\|d + sxd | t x d | 1.7 | 0.12 | 0.94 |
| t + ht **+** s + d + t\|sxd | t x s x d | -1.6 | 0.04 | 0.98 |
| *E) Control vs. 100 nm low* | |  |  |  |
| **t + ht + s + d + sxd** |  | 14.7 |  |  |
| t + ht **+** t\|s + d + sxd | t x s | 18.7 | 0 | 1 |
| t + ht + s + t\|d + sxd | t x d | 18.5 | 0.26 | 0.88 |
| t + ht **+** s + d + t\|sxd | t x s x d | 18.6 | 0.15 | 0.93 |
| *F) Control vs. 100 nm high* | |  |  |  |
| **t + ht + s + d + sxd** |  | -2.5 |  |  |
| t + ht **+** t\|s + d + sxd | t x s | 0.3 | 1.21 | 0.54 |
| t + ht + s + t\|d + sxd | t x d | 1.5 | 0 | 1 |
| t + ht **+** s + d + t\|sxd | t x s x d | 1.5 | 0 | 1 |

Treatment and time until hatching (ht) were entered as fix effects, and sire, dam, and sire x dam were entered as random effects. Model fit was examined as described for Tables S6-7.
